# Features of emotion recognition in deviant adolescents

**DOI:** 10.1192/j.eurpsy.2021.567

**Published:** 2021-08-13

**Authors:** A. Abolonin, S. Gusev

**Affiliations:** Addictive States Department, Mental Health Research Institute, Tomsk, Russian Federation

**Keywords:** Aggression, delinquent adolescents, emotion recognition

## Abstract

**Introduction:**

Some researchers believe that an increased level of aggression and cruelty towards others in delinquent adolescents is due to impaired recognition of emotions and empathy.

**Objectives:**

The aim of our study was to study the recognition of emotions in deviant adolescents.

**Methods:**

As a material, 156 juvenile offenders from 13 to 19 years old were selected who were in the camp for delinquent adolescents “Sibextrem”. All of them committed any offenses, they were registered with the social welfare authorities and the police. The adolescents were trained to reduce aggressiveness. During the training, several exercises were carried out. In the first exercise, the teenagers were asked to identify the emotions depicted in the photographs. In the second, determine what emotional state their peers portray

**Results:**

During the training process, 78% of adolescents could not identify the emotions presented. This was typical not only for the recognition of standard images, but also for the presentation of emotions by peers. As a result of the training, most adolescents, 64.2%, learned to quite accurately recognize nonverbal emotions. As a result, the number of aggressive manifestations decreased by 31.6%. Mutual understanding and communication improved.

**Conclusions:**

The results obtained indicate that deviant adolescents have impaired emotional perception of others. Difficulty in assessing emotions creates tension in interpersonal relationships and can contribute to the manifestation of various forms of aggressive behavior. The vector of research we have chosen shows the need for further study of the emotional sphere of adolescents and its relationship with deviant forms of behavior.

